# Metatranscriptomic profiling uncovers a distinctive pepper virome in tropical Indonesia

**DOI:** 10.3389/fpls.2026.1933649

**Published:** 2026-09-04

**Authors:** Andika Septiana Suryaningsih, Seok-Yeong Jang, Dhayanti Makyorukty, Hee-Seong Byun, Wahyu Ridwan Nanta, Won Kyong Cho, Sun-Jung Kwon, Jang-Kyun Seo

**Affiliations:** 1 Department of International Agricultural Technology, Seoul National University, Pyeongchang, Republic of Korea; 2 Department of Plant Protection, Faculty of Agriculture, IPB University, Bogor, Indonesia; 3 Crop Protection Division, National Institute of Agricultural Sciences, Rural Development Administration, Wanju, Republic of Korea; 4 People’s Coalition for Food Sovereignty, Bogor, West Java, Indonesia; 5 Agriculture and Life Sciences Research Institute, Kangwon National University, Chuncheon, Republic of Korea; 6 Institutes of Green Bio Science and Technology, Seoul National University, Pyeongchang, Republic of Korea

**Keywords:** capsicum annuum, cucumber mosaic virus, Indonesia, pepper virome, RNA-Seq

## Abstract

Pepper (*Capsicum annuum* L.) is an important horticultural crop in Indonesia, yet the virome structure of pepper fields in the country remains largely unknown. Here, we used RNA-Seq-based metatranscriptomics to characterize viruses associated with symptomatic pepper plants from six geographically distinct locations in West Java. Six pooled regional libraries were analyzed, resulting in the identification of 129 plant virus-associated contigs. The pepper virome was strongly dominated by cucumber mosaic virus (CMV), while pepper vein yellows virus (PeVYV) and pepper yellow leaf curl Indonesia virus (PYLCIV) were consistently detected across all regions. Several lower-abundance viruses showed regional patterns, including chilli veinal mottle virus, potato virus Y, pepper cryptic virus 2, PeVYV-associated RNA, and tomato yellow leaf curl Kanchanaburi virus. Phylogenetic analyses suggested that CMV in West Java is related to an established Indonesian lineage, whereas PeVYV and PYLCIV populations are genetically structured across regions. In addition, RNA-Seq revealed two previously unrecognized putative virus-associated sequences related to tobacco bushy top virus and grapevine endophyte endornavirus. Because the libraries were generated from pooled samples, read-based values were interpreted as relative viral RNA abundance rather than field incidence. These findings reveal a CMV-dominated but regionally structured pepper virome in West Java and provide a foundation for virus surveillance and disease management in Indonesia.

## Introduction

Pepper (*Capsicum annuum* L.) is one of the most economically important horticultural crops and contributes substantially to the agricultural economy in Indonesia (Sativa et al., 2017). Despite its economic importance, pepper production in Indonesia is frequently constrained by viral diseases, which can reduce yield, fruit quality, and marketability (Fadhila et al., 2020; Wahyono et al., 2023). Several viruses have been reported in pepper in Indonesia, including cucumber mosaic virus (CMV), pepper yellow leaf curl Indonesia virus (PYLCIV), chili veinal mottle virus (ChiVMV), pepper mild mottle virus (PMMoV), tobacco mosaic virus (TMV), tomato yellow leaf curl virus (TYLCV), potato virus Y (PVY), pepper cryptic virus 2 (PCV2), and pepper vein yellows virus (PeVYV) (Damiri et al., 2018; Fadhila et al., 2020; Helina et al., 2025; Surwadinata et al., 2026; Suryaningsih et al., 2026). Mixed viral infections are also common in pepper and represent a defining feature of virus disease complexes under field conditions (Kim et al., 2014; Jo et al., 2017; Kwon et al., 2018). Such mixed infections complicate disease diagnosis because field symptoms often cannot be attributed to a single causal agent. Moreover, interactions among co-infecting viruses may influence symptom severity, virus accumulation, and host defense responses (Murphy and Bowen, 2006; Kwon et al., 2023). Thus, surveys that focus only on a limited number of target viruses may provide an incomplete understanding of the actual viral disease pressure affecting pepper crops.

RNA sequencing (RNA-Seq)-based metatranscriptomics has become a powerful approach for plant virome analysis (Jo et al., 2017; Rumbou et al., 2020; Sidharthan et al., 2020; Dias et al., 2023). Unlike conventional PCR-based diagnostics, which generally detect only selected known viruses, metatranscriptomic sequencing enables the simultaneous detection of known, divergent, and previously unreported viruses without prior assumptions about their identity (Rumbou et al., 2020; Sukhorukov et al., 2022). This approach can also provide genome-level information useful for analyzing viral diversity, mixed-infection patterns, and phylogenetic relationships. In pepper, in which virus-like symptoms often overlap and plants are frequently co-infected by multiple viruses, RNA-Seq-based virome analysis is particularly valuable for characterizing the viral communities, determining the frequency and composition of mixed infections, and comparing regional patterns of virus diversity and occurrence (Jo et al., 2017; Dias et al., 2023). This information is fundamental to virus disease management because it provides a basis for prioritizing diagnostic targets, monitoring dominant or emerging viruses, and developing integrated control strategies adapted to local production systems.

Indonesia provides a distinctive agroecological and geographic setting for plant virome studies. As an equatorial archipelago, Indonesia has diverse agricultural landscapes, a tropical climate with warm temperatures and high humidity, and year-round cultivation of many horticultural crops (Yamanaka, 2016). In addition, pepper fields in many production areas in Indonesia are located near forest margins or within mixed-cropping systems, creating ecological interfaces among cultivated crops, weeds, wild host plants, and vector communities (Wahyono et al., 2023; Yolanda et al., 2024). These conditions may support continuous virus maintenance, overlapping crop cycles, and persistent populations of insect vectors. Therefore, the viromes of Indonesian pepper fields may have distinctive features shaped by the country’s diverse agroecological conditions.

Although several studies have investigated pepper-infecting viruses in Indonesia using conventional approaches (Damiri et al., 2018; Fadhila et al., 2020; Helina et al., 2025; Surwadinata et al., 2026), no virome-level analysis of pepper fields has yet been reported from the country. In this study, we used an RNA-Seq-based metatranscriptomic approach to characterize the pepper-field viromes in four geographically separated pepper-growing regions (Bogor, Cianjur, Garut, and West Bandung) of Indonesia. We aimed to identify viruses associated with pepper fields in each region, compare regional patterns of virus diversity and occurrence, and examine phylogenetic relationships among the major virus isolates detected across regions. To our knowledge, this study represents the first virome-level analysis of viruses infecting pepper in Indonesia and provides foundational information for improved virus diagnostics, regional surveillance, and integrated management of pepper viral diseases.

## Materials and methods

### Sample collection and RNA-Seq

**Figure 1:**
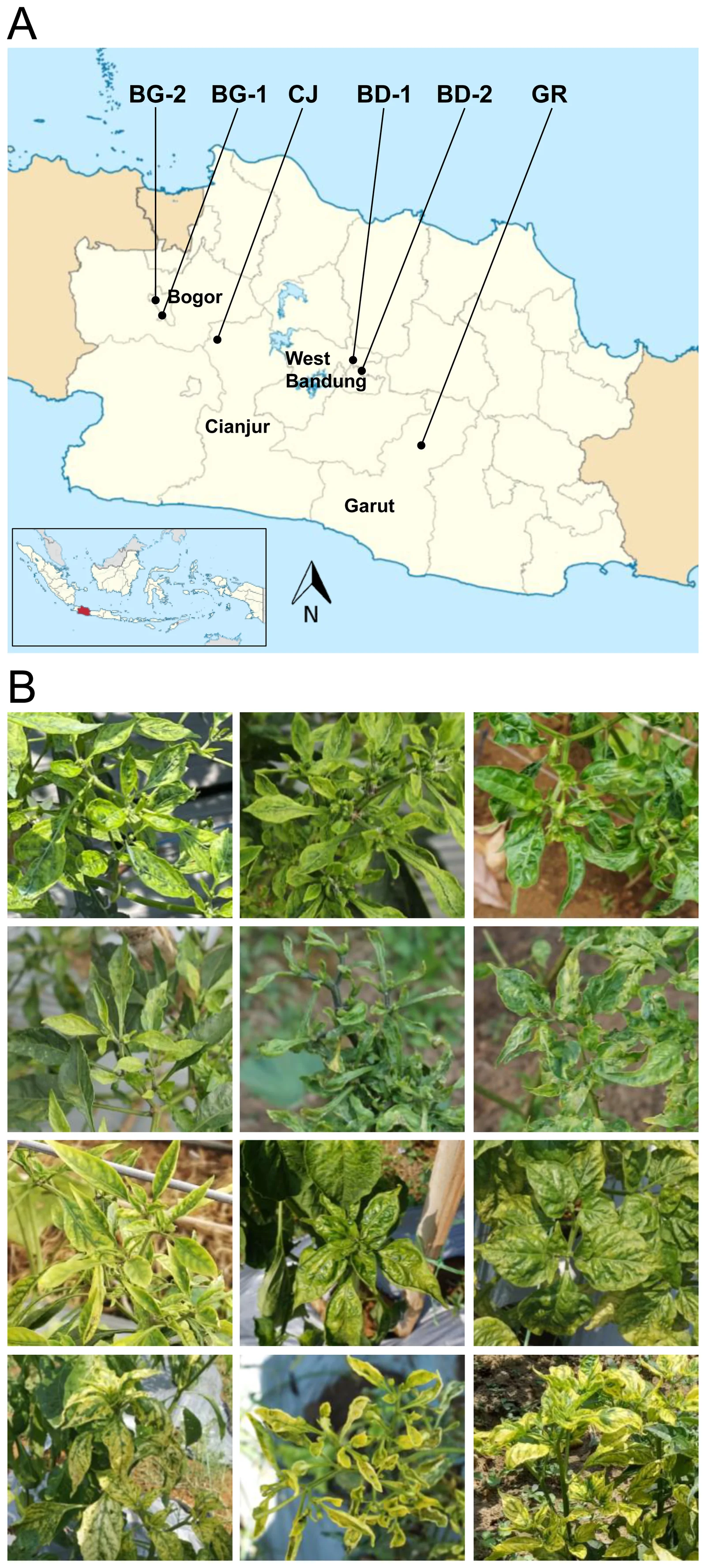
Sampling locations and representative virus-like symptoms of pepper plants collected in West Java, Indonesia. **(A)** Sampling locations in Bogor, Cianjur, Garut, and West Bandung. **(B)** Representative pepper plants showing virus-like symptoms observed in the field, including leaf yellowing, mosaic or mottling, leaf curling, and stunting. BG-1: Situ Gede, Bogor; BG-2: Ciomas, Bogor; CJ: Pacet, Cianjur; GR: Tarogong Kaler, Garut; BD-1: Cikole, West Bandung; BD-2: Lembang, West Bandung.

Field surveys were conducted in major pepper-producing areas of West Java, Indonesia, in August 2024. Pepper (*Capsicum annuum* L.) plants showing typical virus-like symptoms, including leaf yellowing, mosaic, mottling, leaf curling, and/or stunting, were selected for sampling. Six sampling locations were included in this study: two locations in Bogor, one in Cianjur, one in Garut, and two in West Bandung (**Figure 1A**). At each location, two pepper fields were surveyed, and ten symptomatic leaf samples were collected from each field. Because pepper cultivar identity could not be confirmed for all individual plants included in each pooled sample, cultivar information was recorded as “mixed” for each sampling location (**Table 1**). Leaf samples collected from the two fields within each location were pooled to generate a single composite sample for RNA-Seq. The six pooled samples were designated according to their sampling locations as Bogor-1 (BG-1), Bogor-2 (BG-2), Cianjur (CJ), Garut (GR), Bandung-1 (BD-1), and Bandung-2 (BD-2) (**Table 1**). Total RNA was extracted from each composite sample using the Hybrid-R™ RNA Extraction Kit (GeneAll, Korea), following the manufacturer’s protocol. cDNA libraries were constructed using the TruSeq Stranded Total RNA with Ribo-Zero Plant kit (Illumina, USA). A total of six cDNA libraries, corresponding to BG-1, BG-2, CJ, GR, BD-1, and BD-2, were sequenced using an Illumina NovaSeq 6000 platform (Macrogen, Korea). Sequencing was performed in a paired-end format with a read length of 150 bp. The raw RNA-Seq data were deposited in the NCBI SRA database with BioProject accession number PRJNA1492756.

**Table 1:** Information on pepper samples collected in Indonesia for virome analysis.

Sample	Geographical region	Cultivar	Collection year	Single or Pooled	No. of samples
BG-1	Situ Gede, Bogor	Mixed	2024	Pooled	20 from 2 fields
BG-2	Ciomas, Bogor	Mixed	2024	Pooled	20 from 2 fields
CJ	Pacet, Cianjur	Mixed	2024	Pooled	20 from 2 fields
GR	Tarogong Kaler, Garut	Mixed	2024	Pooled	20 from 2 fields
BD-1	Cikole, West Bandung	Mixed	2024	Pooled	20 from 2 fields
BD-2	Lembang, West Bandung	Mixed	2024	Pooled	20 from 2 fields

### Bioinformatic analysis of RNA-Seq data

Raw reads generated from each RNA-Seq library were processed to remove adapter sequences and low-quality bases using BBDuk version 39.01, a component of the BBMap package (https://bbmap.org/). The quality-filtered reads were then assembled *de novo* using Trinity v2.15.0 under default configurations. The resulting contigs were analyzed uisng BLASTn and BLASTx searches against viral nucleotide and protein sequences available in the NCBI GenBank database with an E-value cutoff of 1 × 10^−10^ to identify virus-associated sequences (Seo et al., 2017). Contigs with lower sequence identity were retained only when they showed clear similarity to conserved viral proteins or domains and were supported by additional evidence, such as genome organization, read mapping, RT-PCR confirmation, or phylogenetic placement. Candidate contigs were further curated manually to remove likely host-derived sequences, low-complexity sequences, non-plant virus sequences, and probable contaminants. Trimmed reads were mapped back to the curated virus-associated contigs, and transcript abundance was normalized as fragments per kilobase of transcript per million mapped reads (FPKM). When multiple contigs were assigned to the same virus, their FPKM values were combined to estimate the total FPKM value for that virus. The FPKM value for each virus was then converted to a relative percentage of the total virus-associated FPKM within each library. These normalized values were used to compare viral RNA composition among the six pooled libraries. Because each library was generated from pooled samples, FPKM-based values were interpreted as relative viral RNA abundance within each library rather than as estimates of field incidence or plant-level infection prevalence. Sequencing depth was further assessed by examining read-mapping coverage across curated virus-associated contigs. Trimmed reads were aligned to the curated contigs using BWA v0.7.17 with default parameters, and read abundance was estimated using eXpress v1.5.1. Low-abundance virus-associated contigs were inspected for continuous read coverage and were further validated by RT-PCR followed by Sanger sequencing of overlapping amplicons. The entire procedure of RNA-Seq was performed by Macrogen Inc. (Korea).

### RT-PCR validation of RNA-Seq-detected viruses

Virus-specific RT-PCR assays were performed to confirm selected viruses detected by RNA-Seq. Primer sets used for validation are listed in **Supplementary Table 1**. For each sample, the same total RNA preparation used for RNA-Seq library construction was used for RT-PCR analysis. Total RNA extracted from healthy pepper plants was used as a negative control in the RT-PCR validation assays. Reactions were performed using BCS™ RT-PCR 2X Master Mix (BioCube, Korea), following the manufacturer’s protocol. RT-PCR was conducted under the following cycling conditions: 48 °C for 15 min for reverse transcription and 95 °C for 5 min for initial denaturation, followed by 35 cycles of 95 °C for 30 s, 58 °C for 30 s, and 72 °C for 1 min. A final extension was performed at 72 °C for 5 min. The resulting amplicons were analyzed by electrophoresis on 1% agarose gels prepared in 1× TAE buffer.

### Sequence determination of virus isolates and phylogenetic analysis

To determine the complete genome sequences of the Indonesian isolates of CMV and PeVYV identified in this study, overlapping RT-PCR fragments covering the contigs were amplified from the same total RNA preparations used for RNA-Seq library construction using primer pairs designed based on the respective virus sequences. The resulting amplicons were directly analyzed by Sanger sequencing. The 5′ and 3′ terminal sequences were determined by rapid amplification of cDNA ends (RACE) using the SMARTer RACE cDNA Amplification Kit (Clontech, USA), according to the manufacturer’s instructions. Because the CMV genomic RNAs were amplified from pooled samples, the sequences should be interpreted as dominant regional consensus sequences rather than as complete genomic sets from individual infected plants. The genome sequences of the Indonesian isolates of CMV and PeVYV identified in this study were deposited in GenBank: CMV-BG1 RNA1 (PZ630760), CMV-BG1 RNA2 (PZ630761), CMV-BG1 RNA3 (PZ630762), CMV-BG2 RNA1 (PZ630763), CMV-BG2 RNA2 (PZ630764), CMV-BG2 RNA3 (PZ630765), CMV-CJ RNA1 (PZ630766), CMV-CJ RNA2 (PZ630767), CMV-CJ RNA3 (PZ630768), CMV-GR RNA1 (PZ630769), CMV-GR RNA2 (PZ630770), CMV-GR RNA3 (PZ630771), CMV-BD1 RNA1 (PZ630772), CMV-BD1 RNA2 (PZ630773), CMV-BD1 RNA3 (PZ630774), CMV-BD2 RNA1 (PZ630775), CMV-BD2 RNA2 (PZ630776), CMV-BD2 RNA3 (PZ630777), PeVYV-BG1 (PZ630778), PeVYV-BG2 (PZ630779), PeVYV-CJ (PZ630780), PeVYV-GR (PZ630781), PeVYV-BD1 (PZ630782), and PeVYV-BD2 (PZ630783).

To obtain the complete sequences of PYLCIV detected in this study, total DNA was extracted from the same plant samples used for RNA-Seq analysis using the DNeasy Plant Mini Kit (QIAGEN, Germany), following the manufacturer’s protocol. Full-length DNA-A and DNA-B components were amplified by PCR using component-specific primer pairs designed to anneal to conserved regions of PYLCIV DNA-A and DNA-B, respectively: 5′-TGTCGTCGTTGCCAATGTCTAT-3′ and 5′-CACTGTATCGATGGAACGCTT-3′ for DNA-A and 5’-TATCTTCTTTGTAACAACACTTC-3’ and 5’-GATAGTCTGTACCGTCATGTA-3’ for DNA-B. PCR products of the expected size were purified and directly sequenced by Sanger sequencing. The genome sequences of the Indonesian isolates of PYLCIV identified in this study were deposited in GenBank: PYLCIV-BG1 DNA-A (PZ630784), PYLCIV-BG1 DNA-B (PZ630785), PYLCIV-BG2 DNA-A (PZ630786), PYLCIV-BG2 DNA-B (PZ630787), PYLCIV-CJ DNA-A (PZ630788), PYLCIV-CJ DNA-B (PZ630789), PYLCIV-GR DNA-A (PZ630790), PYLCIV-GR DNA-B (PZ630791), PYLCIV-BD1 DNA-A (PZ630792), PYLCIV-BD1 DNA-B (PZ630793), PYLCIV-BD2 DNA-A (PZ630794), and PYLCIV-BD2 DNA-B (PZ630795).

To confirm the RNA-Seq-derived contig sequences corresponding to the putative umbravirus related to tobacco bushy top virus (TBTV) and the putative alphaendornavirus related to grapevine endophyte endornavirus (GEEV), overlapping RT-PCR fragments covering the assembled contigs were amplified from the same total RNA preparations used for RNA-Seq library construction using primers designed based on the respective contig sequences. The resulting amplicons were directly analyzed by Sanger sequencing. The partial genomic sequences of the putative novel viruses identified in this study were deposited in GenBank: TBTV-like putative umbravirus (PZ630796) and GEEV-like putative alphaendornavirus (PZ630797).

Phylogenetic analyses were performed for the major viruses identified across the six locations, including CMV, PeVYV and PYLCIV. Genome sequences of the representative reference isolates for each virus were retrieved from GenBank (Heo et al., 2020). In particular, given the limited availability of complete PYLCIV DNA-A and DNA-B sequences outside Indonesia and Malaysia, phylogenetic analyses were performed using 21 DNA-A and 21 DNA-B reference sequences, all of which were reported from isolates collected in these two countries. Multiple sequence alignments were generated using ClustalW implemented in MEGA12 (Kumar et al., 2024). Phylogenetic trees were constructed using the maximum likelihood method with the Tamura-Nei model implemented in MEGA12. Branch support was assessed using 1,000 bootstrap replicates, and the resulting trees were visualized using MEGA12. Pairwise nucleotide sequence comparison was performed using the Needle software from the EBI website (https://www.ebi.ac.uk/Tools/psa/emboss_needle/).

## Results

### Generation of RNA-Seq datasets for comparative analysis of Indonesian pepper viromes

Pepper samples showing typical virus-like symptoms were collected from six geographically distinct locations in West Java, Indonesia, in 2024: BG-1, BG-2, CJ, GR, BD-1, and BD-2 (**Figure 1**; **Table 1**). For each location, twenty leaf samples collected from two fields were pooled to generate one composite sample for RNA-Seq analysis, resulting in six regional sequencing libraries (**Tables 1**, **2**). RNA-Seq of the six libraries generated 494,262,684 raw reads in total, with 75,396,484–90,005,734 reads per library. *De novo* assembly yielded 549,876 contigs, ranging from 74,808 contigs in BD-1 to 128,401 contigs in BD-2 (**Table 2**). These data provided sufficient sequencing depth and assembly output for downstream virome analysis.

**Table 2:** Summary statistics of raw RNA-Seq data generated for pepper virome analysis in Indonesia.

Library	Sample	Total reads	Assembled contigs	# of contigs with partial BLASTn matches to the viral DB	# of contigs with partial BLASTx matches to the viral DB	# of contigs identified as plant viral sequences
IDN-1	BG-1	75,396,484	75,460	61	5,945	28
IDN-2	BG-2	85,495,632	79,894	48	5,685	15
IDN-3	CJ	78,486,422	84,362	62	6,253	19
IDN-4	GR	90,005,734	106,951	72	7,422	27
IDN-5	BD-1	78,140,744	74,808	86	6,320	20
IDN-6	BD-2	86,737,668	128,401	129	10,110	20

Similarity searches against viral reference databases identified virus-associated contigs in all six libraries (**Table 2**). The number of contigs with partial BLASTn matches to viral sequences ranged from 48 in BG-2 to 129 in BD-2, whereas BLASTx-based matches ranged from 5,685 in BG-2 to 10,110 in BD-2 (**Table 2**). After further classification, 129 contigs were identified as plant virus-associated sequences across the six libraries. The number of curated plant viral contigs varied among regions, with the highest numbers detected in BG-1 and GR, and the lowest number detected in BG-2 (**Table 2**). Overall, these results indicate that the RNA-Seq datasets were suitable for comparative analysis of pepper-associated viromes and revealed regional differences in virus-derived sequence composition.

### Regional composition of pepper-infecting viruses in West Java based on RNA-Seq analysis

Because each sequencing library was generated from pooled regional samples, read counts and FPKM values were interpreted as relative viral RNA abundance within each library, not as estimates of plant-level infection incidence (**Table 3**). RNA-Seq analysis revealed that CMV was the predominant viral RNA component in all six libraries (**Table 3**). PeVYV and PYLCIV were also consistently detected, indicating that these viruses constituted a shared component of the pepper virome across the surveyed regions. In addition, the RNA-Seq data revealed regional variation in the composition of lower-abundance viruses, suggesting local differences in pepper-associated viral communities in West Java (**Table 3**). To validate the RNA-Seq results, RT-PCR assays were performed for selected major viruses detected in the RNA-Seq data. The RT-PCR results confirmed the presence of the targeted viruses in the corresponding regional samples and showed detection patterns that were largely consistent with the RNA-Seq-based profiles (**Supplementary Figure S1**). These results support the reliability of the RNA-Seq analysis for identifying major pepper-infecting viruses and comparing regional virome composition.

**Table 3:** Viruses detected by RNA-Seq in pepper samples collected from six geographically distinct locations in West Java, Indonesia.

Sample	Virus	Acronym	Read count	FPKM
BG-1	Cucumber mosaic virus	CMV	13091721	278677.09
	Pepper cryptic virus 2	PCV2	72719	2541.26
	Pepper vein yellows virus	PeVYV	59382	2637.80
	Putative umbravirus (tobacco bushy top virus-like)	–	9033	406.97
	Pogostemon alphacytorhabdovirus 1	PogACRV1	6205	22.71
	Pepper yellow leaf curl Indonesia virus DNA-A	PYLCIV DNA-A	5188	96.94
	Pepper vein yellows virus-associated RNA	PeVYVaRNA	4371	75.19
	Pepper yellow leaf curl Indonesia virus DNA-B	PYLCIV DNA-B	1888	68.47
BG-2	Cucumber mosaic virus	CMV	12505113	235944.85
	Chilli veinal mottle virus	ChiVMV	873337	3997.85
	Pepper vein yellows virus	PeVYV	99150	2649.51
	Pepper yellow leaf curl Indonesia virus DNA-A	PYLCIV DNA-A	3013	73.64
	Pepper yellow leaf curl Indonesia virus DNA-B	PYLCIV DNA-B	2545	44.00
	Pepper vein yellows virus-associated RNA	PeVYVaRNA	278	4.42
CJ	Cucumber mosaic virus	CMV	22114075	319912.83
	Pepper vein yellows virus-associated RNA	PeVYVaRNA	491491	5771.37
	Chilli veinal mottle virus	ChiVMV	155646	524.31
	Pepper vein yellows virus	PeVYV	59415	1153.27
	Pepper cryptic virus 2	PCV2	37445	911.66
	Pepper yellow leaf curl Indonesia virus DNA-A	PYLCIV DNA-A	10618	65.6
	Pepper yellow leaf curl Indonesia virus DNA-B	PYLCIV DNA-B	10445	64.54
	Pogostemon alphacytorhabdovirus 1	PogACRV1	3407	21.99
	Capsicum annuum amalgavirus 1	CaAV1	280	2.85
GR	Cucumber mosaic virus	CMV	1354988	29225.51
	Potato virus Y	PVY	297202	1331.37
	Pepper vein yellows virus	PeVYV	48543	1346.28
	Pepper cryptic virus 2	PCV2	23934	754.01
	Pepper yellow leaf curl Indonesia virus DNA-B	PYLCIV DNA-B	15391	321.00
	Tomato yellow leaf curl Kanchanaburi virus DNA-B	TYLCKaV DNA-B	3053	50.82
	Putative alphaendornavirus (grapevine endophyte endornavirus-like)	–	2674	12.25
	Pepper yellow leaf curl Indonesia virus DNA-A	PYLCIV DNA-A	1974	63.48
	Tomato yellow leaf curl Kanchanaburi virus DNA-A	TYLCKaV DNA-A	140	12.95
BD-1	Cucumber mosaic virus	CMV	9093168	191893.39
	Chilli veinal mottle virus	ChiVMV	153984	956.62
	Pepper vein yellows virus	PeVYV	31009	783.74
	Pepper yellow leaf curl Indonesia virus DNA-A	PYLCIV DNA-A	12313	284.77
	Pepper yellow leaf curl Indonesia virus DNA-B	PYLCIV DNA-B	6913	162.62
	Pepper cryptic virus 2	PCV2	4038	185.48
	Pogostemon alphacytorhabdovirus 1	PogACRV1	2590	11.81
BD-2	Cucumber mosaic virus	CMV	11597530	183151.94
	Chilli veinal mottle virus	ChiVMV	439601	1672.24
	Pepper vein yellows virus	PeVYV	145571	2751.32
	Pepper vein yellows virus-associated RNA	PeVYVaRNA	19284	253.28
	Pepper yellow leaf curl Indonesia virus DNA-A	PYLCIV DNA-A	17485	116.1
	Pogostemon alphacytorhabdovirus 1	PogACRV1	1202	7.18
	Pepper yellow leaf curl Indonesia virus DNA-B	PYLCIV DNA-B	538	8.11
	Putative umbravirus (tobacco bushy top virus-like)	–	268	8.18
	Pepper cryptic virus 2	PCV2	212	6.11

**Figure 2:**
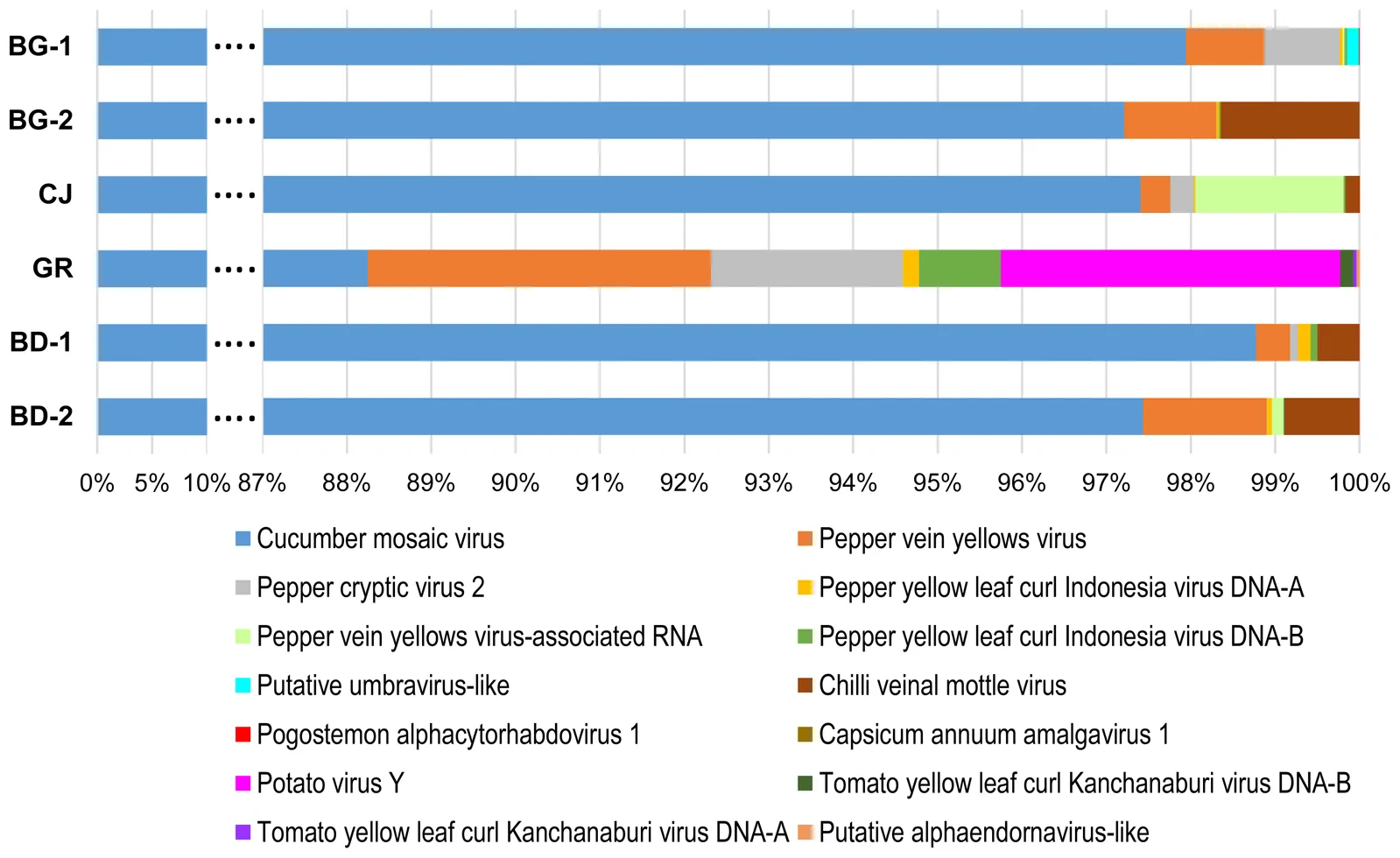
Relative composition of virus-associated RNA signals in pepper samples from six locations in West Java, Indonesia. Virus-associated FPKM values obtained from each RNA-Seq library were normalized to the total virus-associated FPKM value within the corresponding library and expressed as relative percentages. These values represent relative viral RNA abundance in pooled regional libraries. The axis break was introduced to provide full-scale context for CMV dominance while allowing lower-abundance viral components to be visualized in the expanded 87–100% range.

To compare regional virome profiles, virus-associated FPKM values were converted to relative percentages within each regional RNA-Seq dataset. Across the six locations in West Java, CMV was the predominant viral RNA component, accounting for 88.25–98.77% of the total virus-associated FPKM signal (**Figure 2**; **Table 3**). In addition to CMV, PeVYV was detected in all six locations, with relative FPKM values ranging from 0.35% in CJ to 4.07% in GR. PYLCIV DNA-A and DNA-B were also detected across all regions, although their relative abundance was low (**Figure 2**; **Table 3**). Despite this common CMV-dominated profile, the composition of lower-abundance viruses differed among regions. GR showed the most distinct virome structure, with the lowest relative CMV abundance and higher FPKM contributions from PeVYV, PVY, PCV2, and PYLCIV DNA-B. In particular, PVY and tomato yellow leaf curl Kanchanaburi virus (TYLCKaV) DNA-A and DNA-B were detected only in GR (**Figure 2**; **Table 3**). Other viruses also showed uneven regional distributions. ChiVMV was detected in BG-2, CJ, BD-1, and BD-2, with the highest relative abundance in BG-2. PeVYV-associated RNA (PeVYVaRNA) was most prominent in CJ, whereas PCV2 was relatively enriched in GR and BG-1 (**Figure 2**; **Table 3**). In addition to known viruses, two previously unreported viral sequences were identified: a putative umbravirus related to TBTV in BG-1 and BD-2 and a putative alphaendornavirus related to GEEV in GR (**Figure 2**; **Table 3**). These putative viruses are described in detail in subsequent sections.

Together, these results indicate that pepper viromes in West Java share a CMV-dominated core profile while also containing viral components that varied among pooled libraries, suggesting apparent differences in pepper-associated viral RNA profiles across sampling locations. However, it should be noted that our results provide a comparative view of viral RNA abundance and regional virome structure, rather than direct estimates of field incidence or infection prevalence, because these estimates are based on relative FPKM values from RNA-Seq libraries.

### Phylogenetic positions of Indonesian isolates of major pepper-infecting viruses

RNA-Seq analysis identified multiple viruses in pepper samples collected from the six locations surveyed in West Java. Among these viruses, CMV, PeVYV, and PYLCIV were consistently detected in all regions and represented the major shared components of the pepper virome. Therefore, to examine the evolutionary relationships of these viruses and infer their potential epidemiological patterns of spread, we determined the complete genome sequences of regional isolates of CMV, PeVYV, and PYLCIV and performed phylogenetic analyses using geographically diverse reference virus sequences available in GenBank.

**Figure 3:**
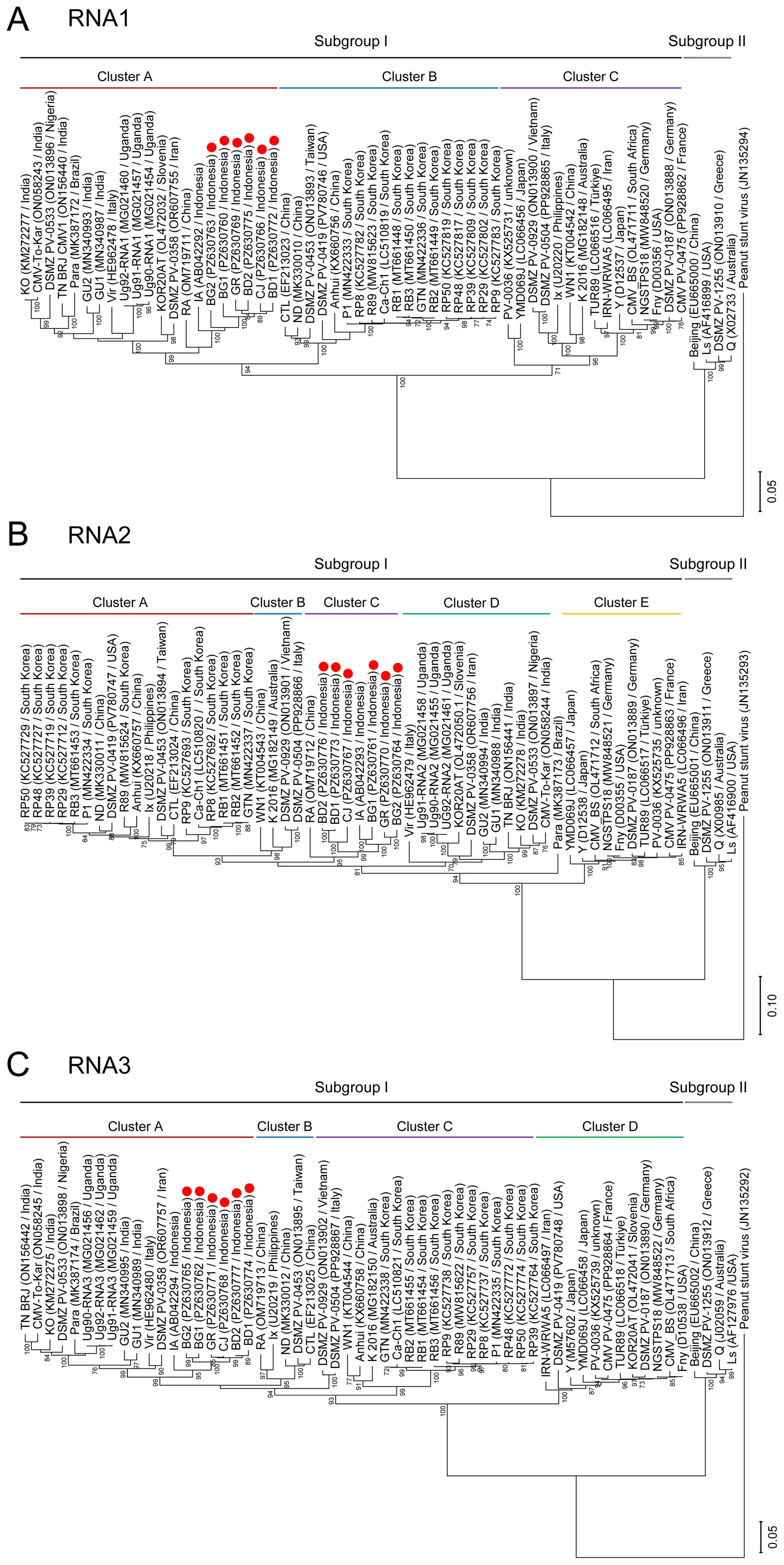
Phylogenetic analyses for the complete genome sequences of RNA1 **(A)**, RNA2 **(B)**, and RNA3 **(C)** of Indonesian cucumber mosaic virus (CMV) isolates, compared with those of representative global isolates. GenBank accession numbers and countries of origin of the analyzed CMV isolates are shown in parentheses after isolate names. Peanut stunt virus was included as an out-group. Phylogenetic trees were reconstructed by the maximum-likelihood method applying the Tamura-Nei model method for nucleotide sequence analyses. Bootstrap values were calculated from 1,000 replicates, and only values greater than 70% are shown. CMV isolates collected from West Java in this study are indicated with red dots.

Phylogenetic analysis of the three genomic RNAs of CMV showed that all six Indonesian CMV isolates collected in this study were closely related to the previously reported Indonesian isolate IA, whose sequence was reported in 2000 (**Figure 3**). This relationship suggests that the dominant CMV sequences detected in the pooled West Java samples are related to an established Indonesian CMV lineage. In the RNA1 and RNA3 trees, Indonesian isolates were positioned closer to isolates reported from India, Uganda, and Iran than to many isolates from nearby East Asian countries such as South Korea, Japan, and China (**Figures 3A, C**). In contrast, RNA2 showed greater phylogenetic differentiation and placed the Indonesian isolates somewhat closer to East Asian isolates than to isolates from India, Uganda, or Iran (**Figure 3B**). Notably, among the foreign isolates, the Chinese isolate RA appeared to be the closest relative to the Indonesian isolates in the RNA1 and RNA2 tree (**Figures 3A, B**).

**Figure 4:**
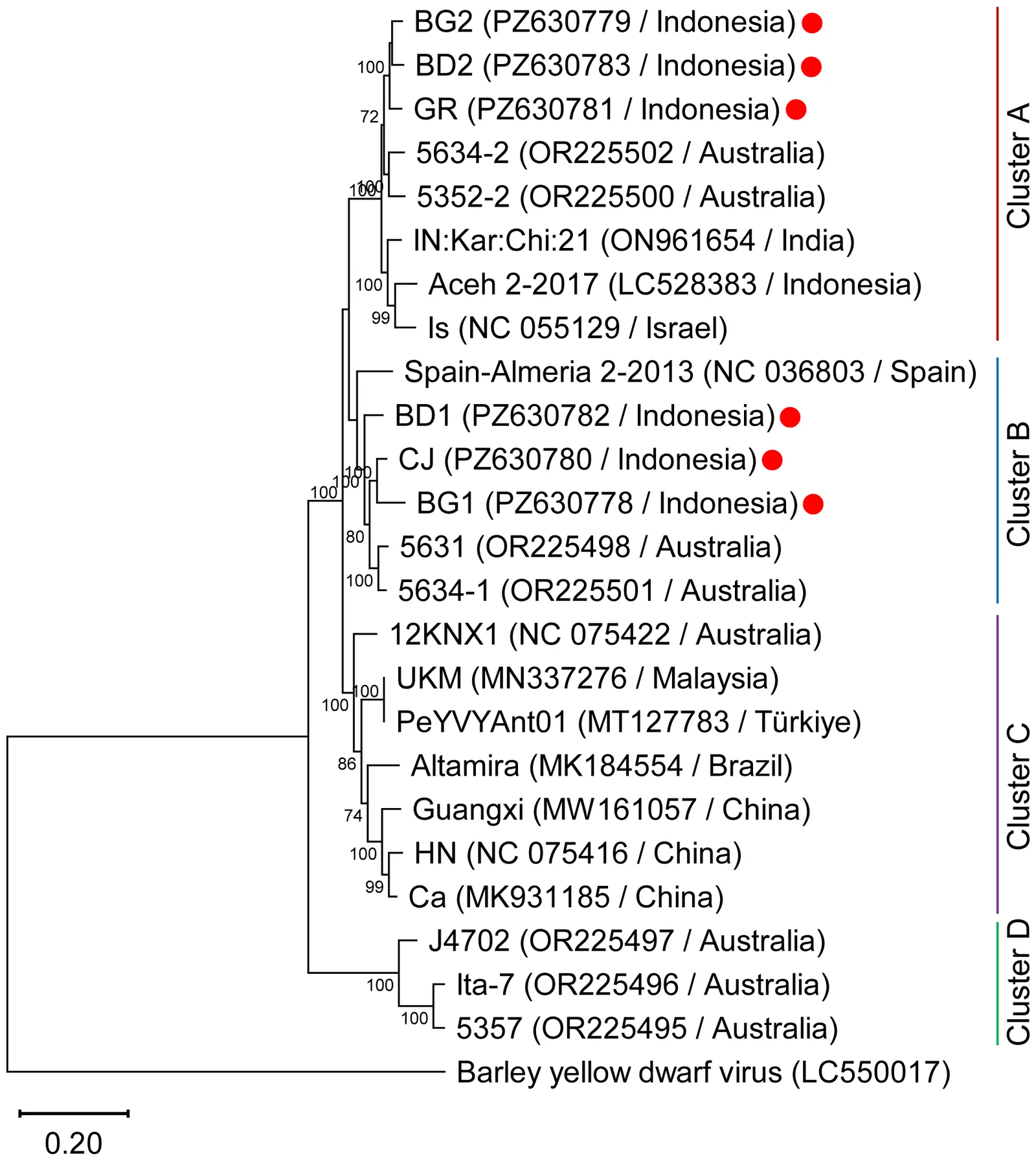
Phylogenetic analysis for the complete genome sequences of Indonesian pepper vein yellows virus (PeVYV) Indonesian isolates, compared with those of representative global isolates. GenBank accession numbers and countries of origin of the analyzed PeVYV isolates are shown in parentheses after isolate names. Barley yellow dwarf virus was included as an out-group. Phylogenetic tree was reconstructed by the maximum-likelihood method applying the Tamura-Nei model method for nucleotide sequence analyses. Bootstrap values were calculated from 1,000 replicates, and only values greater than 70% are shown. PeVYV isolates collected from West Java in this study are indicated with red dots.

Phylogenetic analysis showed that the PeVYV isolates from West Java were divided into two distinct lineages (**Figure 4**). BG-2, BD-2, and GR clustered in cluster A, where they were closely related to Australian isolates 5634-2 and 5352-2. In contrast, BG-1, CJ, and BD-1 grouped in cluster B and showed close affinity to Australian isolates 5631 and 5634-1. The previously reported Indonesian isolate, Aceh 2-2017, which was collected in 2017 from Aceh Province, northern Sumatra, was also placed in cluster A but did not group directly with the West Java isolates (**Figure 4**).

**Figure 5:**
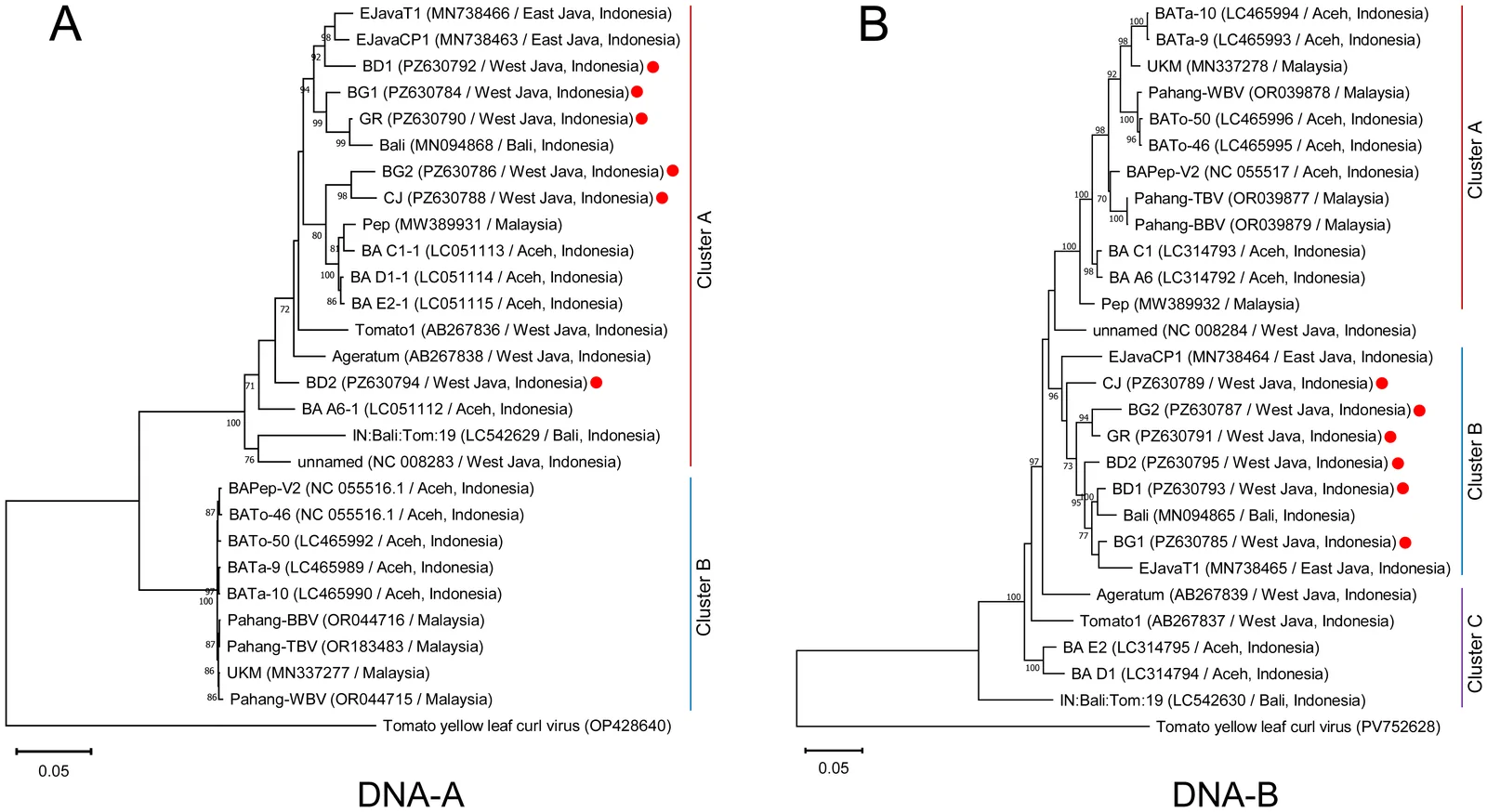
Phylogenetic analyses for the complete genome sequences of DNA-A **(A)** and DNA-B **(B)** of Indonesian pepper yellow leaf curl Indonesia virus (PYLCIV) isolates, compared with those of representative global isolates. The GenBank accession numbers and countries of origin of the analyzed PYLCIV isolates are shown in parentheses after isolate names. For the Indonesian isolates, the collection region of each isolate is also indicated in parentheses. Tomato yellow leaf curl virus was included as an out-group. Phylogenetic trees were reconstructed by the maximum-likelihood method applying the Tamura-Nei model method for nucleotide sequence analyses. Bootstrap values were calculated from 1,000 replicates, and only values greater than 70% are shown. PYLCIV isolates collected from West Java in this study are indicated with red dots.

Phylogenetic analysis of PYLCIV DNA-A and DNA-B showed that the West Java isolates identified in this study were closely related to previously reported Indonesian isolates, particularly those from East Java and Bali (**Figure 5**). Because publicly available complete sequences of PYLCIV DNA-A and DNA-B are largely limited to isolates from Indonesia and Malaysia, the analysis primarily reflects regional relationships within Southeast Asia rather than global patterns of virus movement. The DNA-A tree reveals that PYLCIV DNA-A populations in West Java are connected to a broader Indonesian lineage distributed across Java and Bali (**Figure 5A**). In contrast, several Aceh and Malaysian isolates formed a separate cluster (**Figure 5A**). The DNA-B tree also shows that the DNA-B component detected in West Java is closely associated with a Java–Bali lineage (**Figure 5B**).

### Identification of two putative novel viruses in Indonesian pepper virome

RNA-Seq analysis identified two previously unreported virus-associated sequences in pepper samples: tobacco bushy top virus-like umbravirus sequences in BG-1 and BD-2 and a grapevine endophyte endornavirus-like alphaendornavirus sequence in GR (**Table 3**). Read-mapping analysis showed continuous coverage and sufficient read depth across the assembled contigs (**Supplementary Figure S2**), supporting that these sequences were unlikely to represent sequencing or assembly artifacts. The RNA-Seq-derived contigs were further confirmed by Sanger sequencing of overlapping RT-PCR amplicons spanning the assembled regions. Attempts to determine the 5′ and 3′ terminal sequences by RACE were unsuccessful, likely because of the low abundance of these viruses in the pooled samples (**Table 3**). Thus, in this study, only partial genomic sequences were obtained for the TBTV-like virus from BG-1 (4,130 nucleotides) and the GEEV-like virus from GR (9,509 nucleotides).

NCBI BLASTn analysis showed that the TBTV-like sequence shared the highest nucleotide identity of 75.92% (with 87% query coverage and E-value of 0.0) with TBTV (GenBank accession # KX216406), whereas the GEEV-like sequence showed the highest similarity of 74.25% (with 9% query coverage and E-value of 3e-17) to GEEV (GenBank accession # NC_019493). BLASTx analysis further supported its relationship to GEEV, as the translated GEEV-like sequence showed the highest amino acid identity of 43.05% (with 100% query coverage and E-value of 0.0) to GEEV (GenBank accession # WJN66645). These results suggested that the two sequences might be related to members of the genera *Umbravirus* and *Alphaendornavirus*, respectively, but were genetically distinct from their closest known relatives.

**Figure 6:**
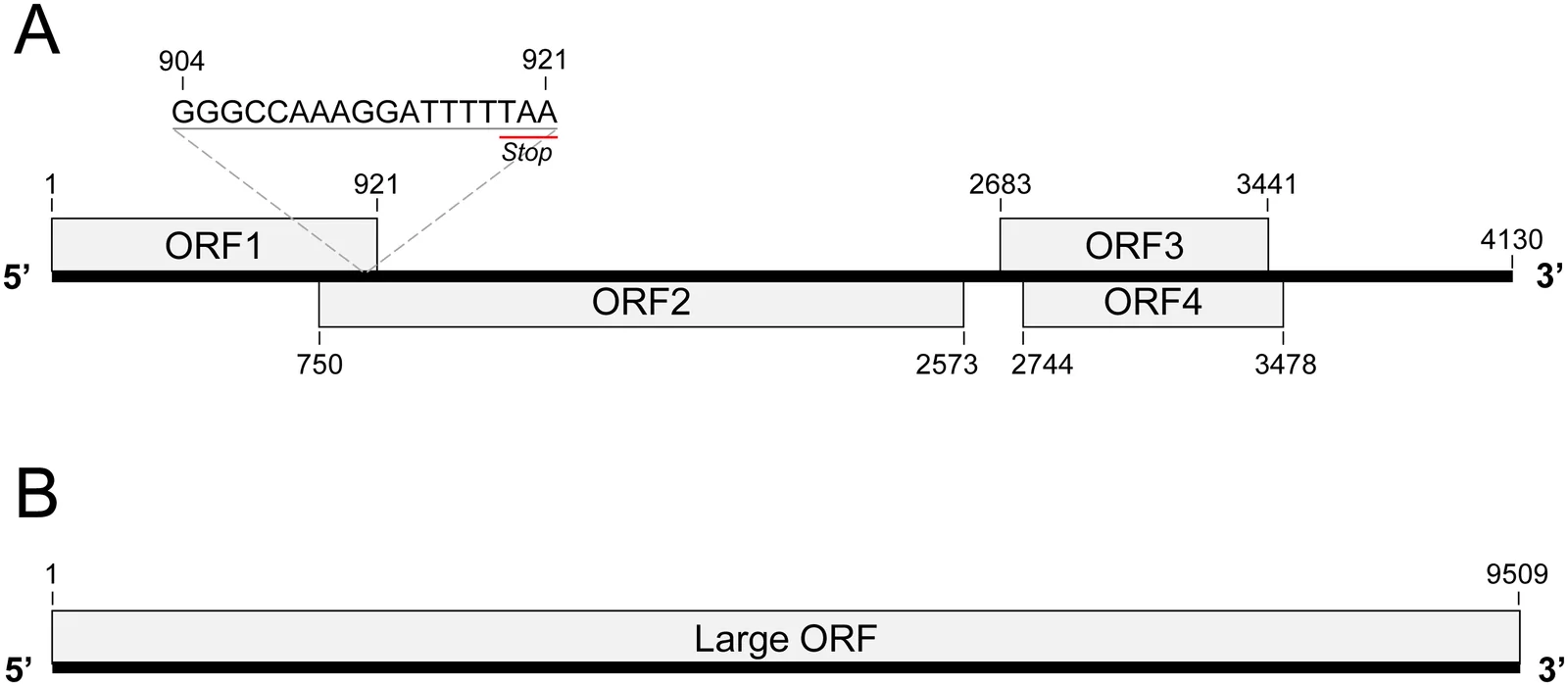
Genome organization of partial sequences of the tobacco bushy top virus (TBTV)-like putative umbravirus and grapevine endophyte endornavirus (GEEV)-like putative alphaendornavirus. **(A)** Schematic representation of the 4,130-nt partial genome sequence of the TBTV-like putative umbravirus, showing four predicted open reading frames (ORFs). A putative umbravirus-like slippery sequence (GGGCCAAAGGATTTTTAA) was identified near the ORF1 termination region, suggesting that ORF1 and ORF2 may be expressed as an ORF1-ORF2 fusion protein through -1 programmed ribosomal frameshifting. **(B)** Schematic representation of the 9,509-nt partial genome sequence of the GEEV-like putative alphaendornavirus. The sequence contains one large partial ORF predicted to encode a polyprotein-like product; however, the 5′ and 3′ terminal regions were not determined.

The partial genome sequence of the putative TBTV-like virus contained four predicted open reading frames, ORF1 to ORF4 (**Figure 6A**). ORF1 encoded a putative protein sharing the highest amino acid identity (54.72%) with the ORF1-encoded hypothetical protein of TBTV. ORF2 encoded a protein containing an RNA-dependent RNA polymerase (RdRp) domain. A conserved umbravirus-like slippery sequence (GGGCCAAAGGATTTTTAA) was identified upstream of the ORF1 termination region, suggesting that ORF1 and ORF2 may be expressed as a 96.3-kDa ORF1–ORF2 fusion protein through -1 ribosomal frameshifting (**Figure 6A**). This predicted fusion protein shared the highest amino acid identity (74.34%) with the TBTV RdRp. ORF3 encoded a putative long-distance movement protein, showing 61.60% amino acid identity to the corresponding TBTV protein, whereas ORF4 encoded a putative cell-to-cell movement protein with 83.88% amino acid identity to the TBTV movement protein. In contrast, the partial genome of the putative GEEV-like virus contained one large partial ORF, whose predicted protein shared the highest amino acid identity (43.05%) with the GEEV polyprotein (**Figure 6B**).

**Figure 7:**
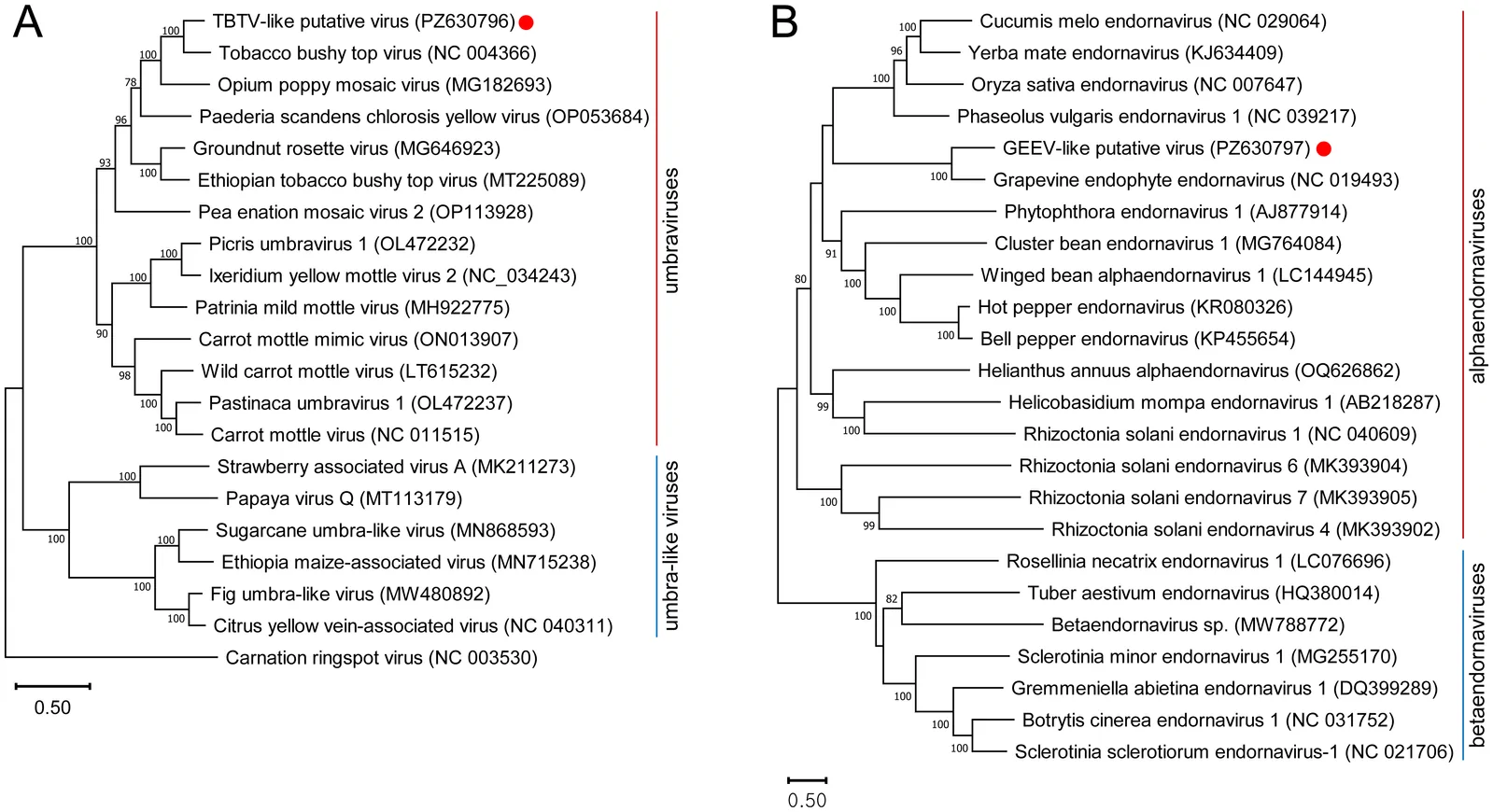
Phylogenetic positions of the TBTV-like putative umbravirus and GEEV-like putative alphaendornavirus identified in this study. **(A)** Phylogenetic analysis based on the amino acid sequence of the predicted ORF1-ORF2 fusion protein of the TBTV-like putative umbravirus and representative umbraviruses and umbra-like viruses. Carnation ringspot virus was included as an out-group. **(B)** Phylogenetic analysis based on the predicted polyprotein amino acid sequence of the GEEV-like putative alphaendornavirus and representative members of the genus *Alphaendornavirus*. Betaendornaviruses were included as an out-group. Virus sequences identified in this study are indicated by red dots. GenBank accession numbers are shown in parentheses. Bootstrap values were calculated from 1,000 replicates, and only values greater than 70% are shown.

Phylogenetic analysis based on the ORF1–ORF2 fusion protein amino acid sequences placed the TBTV-like virus within the genus *Umbravirus* and closest to TBTV (**Figure 7A**). Pairwise sequence comparison using EMBOSS Needle software showed 70.8% nucleotide identity between the TBTV-like sequence and TBTV. Because the ICTV species demarcation criterion for the genus *Umbravirus* uses less than 70% genome-wide nucleotide identity as a threshold for species separation (Ryabov et al., 2012), the TBTV-like virus identified here is best regarded as a divergent TBTV-related isolate rather than a distinct umbravirus species, based on the available partial genome sequence.

Phylogenetic analysis of the polyprotein amino acid sequences placed the GEEV-like virus within the genus *Alphaendornavirus*, with GEEV as its closest known relative (**Figure 7B**). Pairwise comparison showed only 42.9% nucleotide identity between the GEEV-like sequence and GEEV, which is below the ICTV species demarcation threshold of 75% nucleotide identity for alphaendornaviruses (Valverde et al., 2019). Therefore, the GEEV-like virus identified in this study likely represents a putative novel species in the genus *Alphaendornavirus*, pending completion of the terminal sequences and formal taxonomic evaluation.

## Discussion

This study provides the first virome-level characterization of pepper-associated viruses in West Java, Indonesia, using RNA-Seq-based metatranscriptomic analysis. Rather than targeting a predefined set of viruses, this approach allowed the simultaneous detection of dominant viruses, secondary viral components, mixed infections, and previously unrecognized virus-associated sequences. This is particularly important for pepper, in which virus-like symptoms are often nonspecific and mixed infections are common under field conditions (Jo et al., 2017). However, because the analysis was based on pooled regional libraries, the results should be interpreted as comparative viral RNA profiles rather than as direct estimates of field incidence or plant-level prevalence. In addition, because each sampling location was represented by a single pooled library, comparisons among locations are descriptive and do not support formal statistical inference. Therefore, the observed differences among libraries should be interpreted as differences in pooled viral RNA profiles, not as statistically validated regional patterns.

A major feature of the West Java pepper virome was a shared CMV-dominated profile, with PeVYV and PYLCIV also consistently detected across the surveyed regions (**Figure 2**; **Table 3**). This pattern suggests that virus pressure in these pepper fields is shaped primarily by a combination of aphid-transmitted RNA viruses and whitefly-transmitted begomoviruses. Although insect vectors were not surveyed in this study, the predominance of CMV is epidemiologically reasonable given its broad host range and nonpersistent transmission by multiple aphid species, which can facilitate rapid movement among pepper, other cultivated crops, weeds, and reservoir hosts. In tropical agroecosystems such as those in Indonesia, year-round cultivation, continuous availability of susceptible hosts, and persistent vector activity may further promote the maintenance and repeated spread of CMV in pepper fields.

The regional differences observed within West Java indicate that the pepper virome is not uniform across production areas. Although CMV, PeVYV, and PYLCIV appear to represent core viral components, lower-abundance viruses varied among locations (**Figure 2**; **Table 3**). This suggests that local factors, including cropping history, cultivar composition, surrounding vegetation, and vector populations, may influence the structure of pepper-associated viral communities. From a disease management perspective, these findings support a surveillance strategy that includes core diagnostic targets such as CMV, PeVYV, and PYLCIV, while also allowing for the detection of regionally enriched viruses.

The virome profile detected in West Java differs from pepper virus complexes reported in several other Asian countries. In Korea, broad bean wilt virus 2 (BBWV2), together with CMV, has been reported as a major viral component of pepper disease complexes during the past decade, and mixed infections involving CMV and BBWV2 are frequently associated with severe disease symptoms (Kim et al., 2014; Kwon et al., 2018, 2023). Tomato spotted wilt virus (TSWV) has also caused serious regional outbreaks in Korean pepper production (Lian et al., 2013; Kwon et al., 2021, 2022). In China, although the dominant pepper-infecting viruses vary markedly among production regions, a large-scale survey in Yunnan Province revealed that TSWV, PeVYV, and CMV were the three most frequently detected viruses in pepper (Li et al., 2021). In contrast, TSWV and BBWV2 were not detected by RNA-Seq in the West Java pepper samples analyzed in this study (**Table 2**). Additional surveys with larger sample sets, broader geographic coverage, and seasonal replication will be needed to determine the factors underlying these differences in virus composition. Nevertheless, the absence of TSWV and BBWV2 from the West Java RNA-Seq libraries suggests that these viruses were either absent or not yet widely established in the surveyed pepper production areas. This finding is epidemiologically important because TSWV can cause severe disease in pepper, whereas BBWV2 may exacerbate disease severity through synergistic interactions in mixed infections with CMV, which was the dominant virus detected in West Java (Kwon et al., 2021, 2023). Continued surveillance for TSWV and BBWV2 is therefore warranted to support early detection and timely management, which will be essential for preventing their establishment and limiting regional spread.

The phylogenetic analyses provide additional insight into the possible origins and movement of the major viruses detected in this study. The close relationship between the current CMV isolates and the previously reported Indonesian isolate IA suggests that the CMV isolates detected in West Java in 2024 may have originated from a pre-existing Indonesian CMV lineage rather than from a recent independent introduction (**Figure 3**). Furthermore, the clustering of the Indonesian isolates from geographically separated pepper-growing regions further indicates that this lineage has likely been maintained locally and dispersed within Indonesian pepper production systems over time. At the same time, in the RNA1 and RNA3 trees, the Indonesian isolates were positioned closer to isolates reported from India, Uganda, and Iran than to many isolates from geographically closer Asian countries, including China, Japan, and South Korea (**Figures 3A, C**). This pattern indicates that the evolutionary history of Indonesian CMV is not explained simply by geographic proximity. Instead, it is more consistent with an older introduction of a subgroup I lineage related to broader tropical or subtropical CMV populations, followed by long-term local maintenance and spread within Indonesian agroecosystems. The distinct placement of RNA2 relative to RNA1 and RNA3 suggests that the Indonesian CMV population may retain different evolutionary signatures among its genomic RNAs, possibly shaped during an earlier phase of CMV introduction into and establishment in Indonesia, rather than reflecting a recent independent introduction (**Figure 3B**).

PeVYV showed a different epidemiological pattern. The separation between West Java isolates and the previously reported Aceh isolate from northern Sumatra further suggests that the PeVYV isolates currently detected in West Java are unlikely to represent a simple geographic extension of the previously reported Aceh lineage (**Figure 4**). Rather, PeVYV in Indonesia appears to comprise multiple genetically distinct lineages distributed across different islands and production regions. The close phylogenetic relationships between West Java isolates and Australian isolates in both clusters A and B suggest that PeVYV populations in Indonesia may be connected to broader regional lineages rather than representing a single, locally confined population (**Figure 4**). It is also likely that PeVYV diversity in Indonesia may have been shaped by multiple introduction events and independent regional diversification after establishment.

PYLCIV represents another important component of the Indonesian pepper virome. Unlike CMV and PeVYV, which have broad international distributions, PYLCIV has been reported mainly from Indonesia and Malaysia, limiting the global context available for phylogenetic comparison (Fadhila et al., 2020). Even within these restricted populations, the PYLCIV trees suggest regional differentiation among Indonesian virus populations (**Figure 5**). The close relationship among West Java, East Java, and Bali isolates is consistent with movement within connected solanaceous crop production systems across Java and nearby islands (**Figure 5**). In contrast, the separation of several Aceh and Malaysian isolates suggests that PYLCIV populations have become genetically structured across provinces and islands (**Figure 5**).

Pogostemon alphacytorhabdovirus 1 (PogACRV1) was also detected at low relative abundance in several pooled libraries (**Figure 2**; **Table 3**). Although it was not a dominant component of the pepper virome, its detection is noteworthy because PogACRV1 has previously been reported from patchouli, black pepper (*Piper nigrum*), and tropical soda apple (*Solanum viarum*) (Bejerman et al., 2023). Its detection in pepper suggests that PogACRV1 may occur across diverse crop and non-crop hosts in tropical agroecosystems, which could be relevant to virus maintenance in mixed-cropping landscapes. However, because no individual-plant testing or vector survey was conducted for PogACRV1 in this study, its association with pepper infection, transmission biology, and contribution to disease symptoms remain unclear. Further studies are needed to clarify its host range, distribution, and epidemiological significance in pepper production systems.

The detection of previously unreported TBTV-like and GEEV-like virus-associated sequences further demonstrates the value of virome analysis in Indonesian pepper fields (**Figure 6**; **Table 3**). These sequences were not the dominant components of the libraries, but their confirmation by read mapping and RT-PCR-based sequencing supports their presence in the samples (**Supplementary Figure S2**). Their biological roles remain uncertain. The TBTV-like sequence appears to represent a divergent TBTV-related umbravirus rather than a clearly distinct new species based on the available partial sequence, whereas the GEEV-like sequence is more consistent with a putative novel alphaendornavirus (**Figure 7**). Because endornaviruses and umbraviruses can have complex biological associations with plants, fungi, or mixed infections, additional work is needed to determine their true host, distribution, transmissibility, and possible contribution to disease.

Overall, this study shows that the pepper virome in West Java differs from virus profiles reported for pepper in several other Asian production regions. The West Java pepper virome was characterized by a CMV-dominated community, consistent detection of PeVYV and PYLCIV, regionally structured secondary viral components, and the presence of previously unrecognized virus-associated sequences. These findings can establish a molecular baseline for pepper virus surveillance in Indonesia and provide a foundation for developing diagnostic and management strategies adapted to local pepper production systems. Future studies involving individual-plant testing, biologically replicated field sampling, and seasonal surveys will be needed to estimate infection incidence and validate the apparent spatial structuring of pepper-infecting viruses in West Java.

## Data Availability

The datasets presented in this study can be found in online repositories. The names of the repository/repositories and accession number(s) can be found below: https://www.ncbi.nlm.nih.gov/, PRJNA1492756.
