# Supplementary material for: Metatranscriptomic profiling uncovers a distinctive pepper virome in tropical Indonesia
Source: Front Plant Sci. 2026 Sep 4;17:1933649. doi: 10.3389/fpls.2026.1933649 (PMC13585761; doi:10.3389/fpls.2026.1933649)
Supplement: Supplementary file 1 [file DataSheet1.pdf]

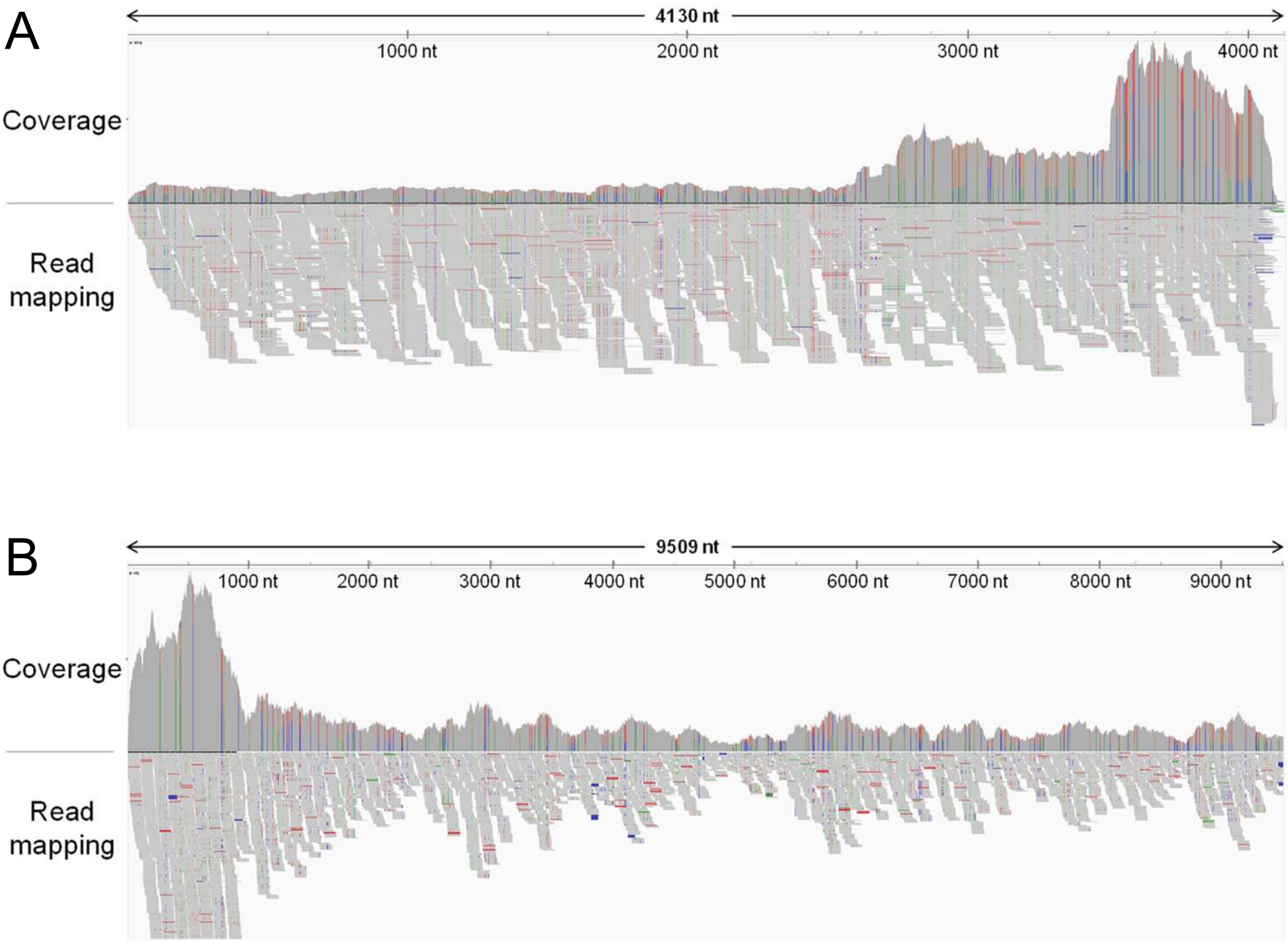

Supplementary Fig. S2. Read coverage and read mapping of contigs corresponding to the TBTV-like putative umbravirus (A) and GEEV-like putative alphaendornavirus (B). RNA-seq reads were mapped onto the assembled contig sequences corresponding to the TBTV-like putative umbravirus (A; 4,130 nt) and the GEEV-like putative alphaendornavirus (B; 9,509 nt). Upper tracks show read coverage across each contig, and lower tracks show individual mapped reads. Read mapping was performed using BWA with default parameters and visualized using Tablet.
