# Supplementary material for: Metatranscriptomic profiling uncovers a distinctive pepper virome in tropical Indonesia
Source: Front Plant Sci. 2026 Sep 4;17:1933649. doi: 10.3389/fpls.2026.1933649 (PMC13585761; doi:10.3389/fpls.2026.1933649)
Supplement: Supplementary file 2 [file DataSheet2.pdf]

Supplementary Table S1. Primers used for RT-PCR validation of RNA-Seq-detected viruses.

Primer	Primer sequence (5’ to 3’)	Purpose
CMV-det-F	CAACAGTCCTCAGCGGCTACGT	To detect cucumber mosaic virus
CMV-det-R	AATTGCGACTTCAACAGGCGAGC	
PeVYV-det-F	GTCGAGGTTTCAGGACAACTGGA	To detect pepper vein yellows virus
PeVYV-det-R	GTGCGACCACAACGACAGGG	
PYLCIV-det-F	ATAGACGCCATTCTGCGCCTGAG	To detect pepper yellow leaf curl Indonesia virus
PYLCIV-det-R	TTCCCACTATCTTCCTCTGCAATC	
ChiVMV-det-F	ATGGAAGTACCCCAAGTTCGAGAC	To detect chili veinal mottle virus
ChiVMV-det-R	CCTACCCTACCGTCCAGTCCG	
PCV2-det-F	GCGACACCAGTTAGTGACACAC	To detect pepper cryptic virus 2
PCV2-det-R	GTTGTTGTGCGCCTACCTGGTG	
PVY-det-F	CCAACTGTGATGAATGGGCTTATG	To detect potato virus Y
PVY-det-R	CAACAACAACAACCCGGCACT	
