# Supplementary material for: Metatranscriptomic profiling uncovers a distinctive pepper virome in tropical Indonesia
Source: Front Plant Sci. 2026 Sep 4;17:1933649. doi: 10.3389/fpls.2026.1933649 (PMC13585761; doi:10.3389/fpls.2026.1933649)
Supplement: Supplementary file 3 [file DataSheet3.pdf]

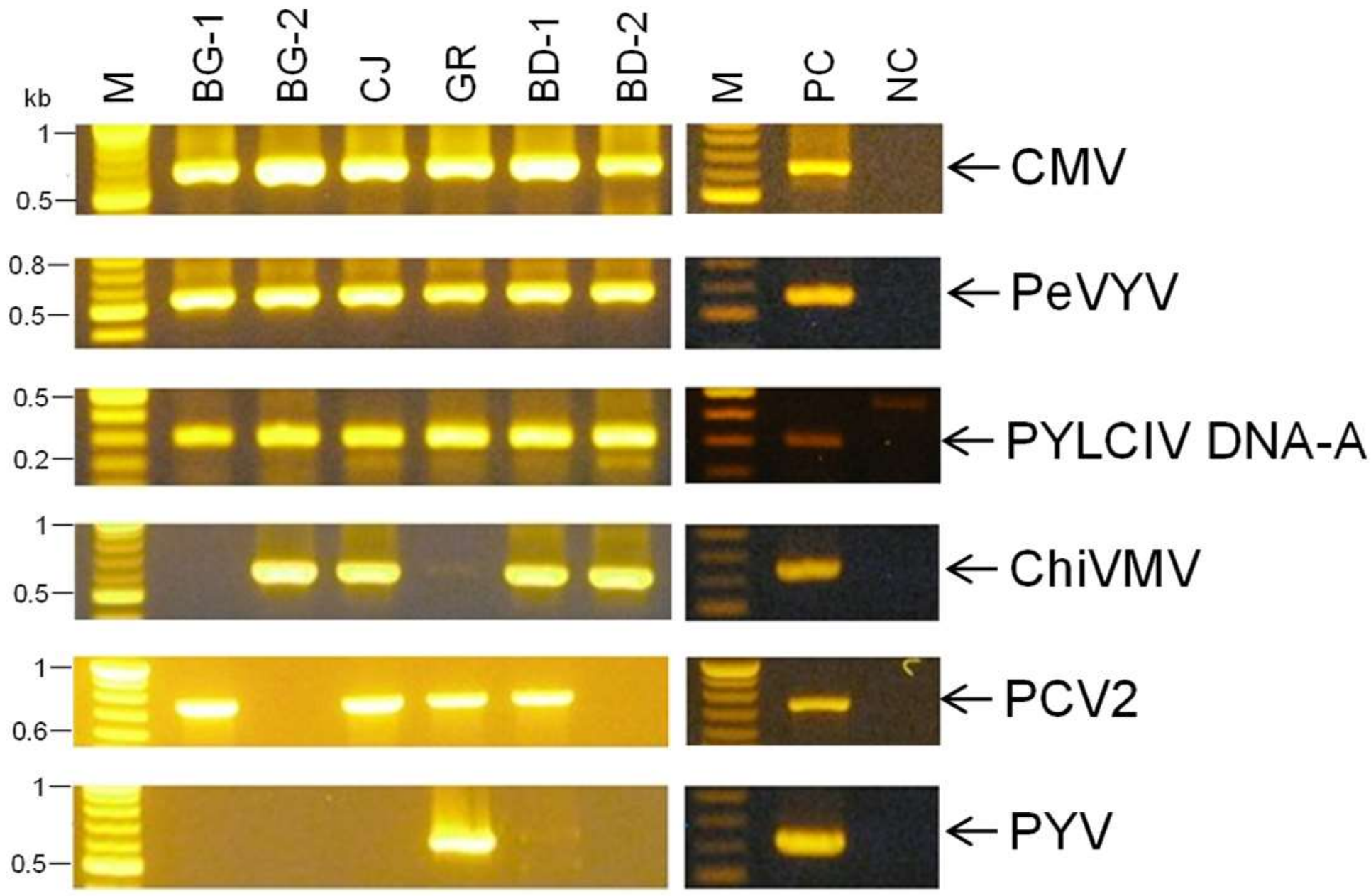

Supplementary Fig. S1. RT-PCR validation of selected viruses detected by RNA-seq. Virus-specific RT-PCR assays were performed to confirm selected viruses detected in the RNA-seq datasets, including CMV, PeVYV, PYLCIV DNA-A, ChiVMV, PCV2, and PVY. The same total RNA preparations used for RNA-seq library construction were used for RT-PCR analysis. Amplified products were separated by electrophoresis on 1% agarose gels in 1x TAE buffer. M, DNA size marker; PC, positive control; NC, negative control. Primer sets used for validation are listed in Supplementary Table S1.
